# Observations on Remittent Fever, Founded upon Cases Observed in the Pennsylvania Hospital

**Published:** 1841-04-10

**Authors:** 


### BIBLIOGRAPHICAL NOTICE.

*Observations on Remittent Fever, founded upon Cases observed in the Pennsylvania Hospital.* By THOMAS STEWARDSON, M. D., one of the Physicians to the Institution. [*American Journal of Medical Sciences.*]

Remittent fever is one of the most frequent diseases; and either in its genuine form, or in the innumerable shades of resemblance by which it approaches to the intermittent type, is met with in the summer and winter of every year. There is no portion of the United States, except New England, which is absolutely exempt from it; but it prevails to a very different degree of intensity; and, like most endemic diseases, is not only modified by situation, but by the peculiar character of the epidemic of the year, and of the series of years. Thus the disease was extremely prevalent in the neighbourhood of Philadelphia in the years 1820–1–2 and gradually became less and less common until the years 1827 and '28, when the epidemic series of years may be said to have ceased. Within the last twelve years it has varied in prevalence and intensity, but it has not prevailed to any great degree in Eastern Pennsylvania, except in some localities, such as the neighbourhood of certain rivers and their tributaries, especially the Susquehanna and Schuylkill. Cases do occur to some extent in other situations, but they are in general mild, and oftener simple intermittents than of the true remittent character. There are, therefore, few fatal cases of the disease at Philadelphia, except amongst strangers, who come from parts of the country where remittents are prevalent, and where they have contracted the fever; and for this reason the cases of severe forms of remittent are of late years more numerous at the Pennsylvania than at the Philadelphia Hospital, because a large number of seamen are admitted into the former institution. These patients contract the disease on the marshy coast of North Carolina and Virginia, and enter the hospital as soon as they reach port. Some of them have suffered from privation and neglect, and die almost immediately after their entrance, which gives a disproportionate number of fatal cases.

The present memoir is relative to the pathological anatomy of these fevers, chiefly as observed in the year 1838, in which the number of fatal cases was remarkably great. Dr. Stewardson was familiar with the pathological anatomy of typhoid fever, which he had studied at Paris, and admits the differences which had been ascertained to exist between it and typhus fever, and remittent fever. In both of these latter diseases it is proven that no lesion of the glands of Peyer exists, and the difficulties which stood in the way of reconciling the discordant testimony of English and French writers upon these diseases are removed. (See American Journal, 1835, and February and August, 1837, papers on Typhus Fever, &c.)

The subject of remittent fever was, however, better known to the English than to French physicians; and it would not have been necessary to have proven that remittent fever was not accompanied with the particular lesions of typhoid fever, had not the fact been assumed without foundation by some of the French pathologists. The experience of English physicians, as well as of American, agreed in admitting that the liver, stomach, and spleen, were the organs most affected. In our own observations we do not remember a case in which two of these organs at least were not *obviously* altered; generally the three were more or less inflamed or congested. As far as the stomach was concerned, the alteration was obviously inflammatory; as to the spleen, it seemed to be simple congestion, now and then taking an inflammatory action. The liver, however, was long known to be the most important cause of many modifications impressed upon the disease; and we have been in the habit of considering its alteration as the result of acute or chronic congestion, or sub-inflammation; rarely is the inflammation carried to the phlegmonous point; almost never, indeed, in our climate. These alterations, it is shown, are accompanied by a peculiar modification of the blood; it is true that objections may be made to so vague a term; but while its imperfection is admitted, the fact exists, and to a certain extent the nature of the alteration may be pointed out. The blood is thin and watery, with a large proportion of serum, and but little altered as to its colour, except that it is much paler than usual; this change takes place not only in the true remittent fever, but in the intermittent, which passes into the former by insensible shades.

Our pathological knowledge was at this point then; but we had no one lesion strikingly characteristic of the disease; it was a compound lesion, produced apparently in great part, if not entirely, by the state of the blood, and apparently dependent mainly upon it. This was especially the case with the parenchymatous organs, the liver and spleen. Dr. Stewardson has gone further; and from the facts which he has observed comes to this conclusion, that there is an alteration of the liver of a peculiar kind, different from what is found in other diseases, and especially characteristic of remittent, and perhaps intermittent. Any one who is acquainted with the character of the author as an observer and pathologist, or who looks merely to the intrinsic evidence of these cases, will see at once that the pathological anatomy is carefully described, so that the only point is to ascertain whether further observation will confirm the conclusions of Dr. Stewardson, as applied to the disease in general. There is no doubt, however, that results which have been uniformly found correct in the cases he has described, will vary but little, if at all, in other cases, and we therefore do not hesitate to regard his researches as highly interesting to the pathologist. If we should speculate on the cause of the bronzed or olive colour of the liver, we would ascribe it to the infiltration, or intimate combination of the bile with the acini, or minute structure of the organ.

A case will illustrate the description of the lesions.

Benjamin Southwick, a sailor, aged twenty-nine, arrived at Philadelphia on the last of August, 1838, four days from Savannah, Georgia. Three days before his arrival he had been exposed in a heavy gale accompanied with much rain, and his clothes wet through. He was taken on the evening of the 31st of August, having previously felt heavy in his head, with a chill, accompanied by pain in the head and back. The chill was slight, did not last long, and during the night he perspired a good deal. On the following day, towards evening, he felt somewhat relieved, but the next day, September 2d, he had a severe chill, followed by high fever, great heat and thirst. On the 3d he was admitted into the Hospital. At that time his skin was hot and dry; pulse frequent, tense and full; headache, stupor, and injection of eye; great tenderness at epigastrium; bowels had been frequently opened. He took the effervescing draught, cold was applied to the head and six cups to the back of the neck.—Low diet.

*September 4th.*—Slept well last night; mind clear, no dulness; senses perfect; no noise in ears; no headache; some yellowness and injection of conjunctiva; a little moisture about forehead; skin warm and slightly yellow; pulse 72, moderately full and strong; no cough; tongue red, moist at edges, dryish in centre, with a whitish fur posteriorly; no appetite; vomiting of a greenish fluid; oppression and some pain across hypochondria, which are tender on pressure; belly generally supple, no meteorism; bowels open twice this morning. ℞.—Cups to epigastrium; cold barley water for drink.

*Evening.*—High fever; skin hot and dry; tongue dry; frequent vomiting; no stupor or delirium. He was directed to take iced drinks and the effervescing draught; and to have a mustard poultice applied to the stomach; also calomel, gr. ½; opii. gr. 1-6th, every two hours.

*5th.*—Has taken four of the pills of calomel and opium. Head hot; some stupor; no delirium or headache; eyes injected; hearing good; respiration frequent and laboured; percussion of chest and respiratory murmur natural; tongue dry and hard; irritability of stomach diminished; no vomiting to-day; little or no tenderness at epigastrium; abdomen distended, tympanitic; bowels opened slightly once this morning; skin warm and slightly moist; yellowness very marked, and of a deep shade; pulse full, moderately strong and a little jerking. The cold drinks and the cold to head were continued, and the following powder directed, viz.: calomel and jalap, *aa* gr. x; cups to head if cephalic symptoms are not relieved.

*Evening.*—Stupor continues; some incoherence; great tenderness at epigastrium; does not complain of sensation of heat there; tongue dry, chapped and smooth; pulse more full. Head to be shaved and cold applied; cold drink as before; also a table-spoonful every hour of the following mixture, viz.: citrate of potash, ʒiij. sulphate of magnesia, ʒv. water, &c., ℥viij.

*6th.*—Dozed during the night but had no sound sleep; did not talk in his sleep; slight wandering but no positive delirium; answers questions readily and without hesitation; no headache; no noise in ears; senses perfect; pupils small, conjunctiva very yellow; slight bleeding at nose; respiration scarcely 20 in the minute, full; oppression at præcordium; great tenderness at epigastrium, which is distended and resonant from gas; has thrown up his medicine several times; the matter last vomited of a light green colour; bowels opened once by injection; stool yellowish brown; skin supple, of natural temperature, of a decided yellow colour; pulse 84, less full and strong; patient lies on his back, quiet. Continue cold drinks and repeat mustard plaster to legs; a blister to the epigastrium. Discontinue the mixture of citrate of potash, and take blue mass, gr. iij.; Rhei. gr. vi. one every four hours.

*Evening.*—The blister has drawn; oppression at præcordium increased; respiration laboured; abdomen distended, tympanitic; mind depressed, slightly wandering; hearing good. The cold drinks were continued and an ounce of tinct. of assafœtida directed by injection.

*7th.*—Eyes very yellow and pupils contracted; oppression at præcordium continues; vomiting of a light greenish fluid; skin warm, deep yellow; pulse 100 to 106, more corded and less full; has taken but two of the pills ordered yesterday, as they irritated the stomach. Discontinue pills and repeat assafœtida injection and cold drinks.

*8th.*—Much the same; great dejection of mind with slight stupor, but no delirium; hearing good; no subsultus; lies on back; yellow colour of skin still deeper than before; hiccup occasionally yesterday and this morning, on which account an injection of a few drops of oil of amber have been administered. Ordered cold drinks with ice; repeat oil of amber if hiccup returns.

He died between 11 and 12 o'clock on the same night; his consciousness being perfect to the last.

*Autopsy* 17 hours after death. *Head.*—Membranes easily detached from the surface of the convolutions; no infiltration under the arachnoid; considerable deep injection of pia mater, especially between the convolutions, soon becoming bright upon exposure to the air; on the upper middle surface of the right hemisphere there is a red patch about the size of half a dollar, owing apparently to a slight effusion of blood into the cells of the pia mater; between two of the convolutions and imbedded in pia mater were found two rounded bodies about the size of small peas, of a yellowish white colour, which which when cut into presented a cavity filled with a friable yellowish white substance; substance of brain firm and of natural colour; cortical substance very distinct; medullary presents a moderate number of red dots on its cut surface; central portions not at all softened; surface of ventricles of a decided yellow colour; no fluid in them; thalami and corpora striata natural; nothing else remarkable; cerebellum natural.

*Chest.*—No adhesions; both lungs supple and crepitating without tubercles or other organic lesion; when cut into, the upper lobes present a yellowish tinge, and when squeezed a spumous fluid of the same colour issues from them in small quantity, otherwise natural; lower lobes of a deep reddish brown, containing a large quantity of red spumous fluid, not at all granulated; texture rather firm, but natural. Heart rather large, but otherwise natural; valves supple, of a yellow colour as well as aorta, which is bright yellow.

*Abdomen.*—Liver of a natural size, flabby, of a bronze colour, which becomes livid in the small lobe; internally of a uniform light bronze colour; acini distinguishable by a slight elevation, but no difference of colour in the two substances; gall bladder filled with a very dark almost black bile, perfectly fluid and thin; a very thin layer of it showing an orange colour. *Spleen* four or five times its natural size, very much softened, pultaceous, brownish black, looks somewhat like clotted venous blood. *Stomach* rather contracted, containing a small quantity of a thin dark coloured fluid. Mucous membrane thrown into folds along the great curvature; its colour, except for a few inches near the pylorus, was bister with a tinge of olive, especially along the great curvature, and with the intermixture of a red tinge in the great cul-de-sac; where the alteration of colour exists, it is softened, friable, and with difficulty raised from the submucous tissue to which it seems to be more adherent than natural; its thickness not materially altered; near the pylorus, where it is pale, it is easily raised into long strips, and is of natural thickness. The mucous membrane of the *duodenum* a little softened, pale, with a slight greenish tinge, otherwise natural; glands of Brunner numerous in same part. The mucous membrane of the *ileum* was also generally pale, with a decided greenish tinge, and some red injection of vessels towards its lower part for the space of a few inches; its thickness was natural; no softening; is easily raised in strips. The glands of Peyer distinct and perfectly natural. Jejunum and upper part of ileum not opened; large intestine healthy; kidneys not examined.

The following description of the alteration of the liver is given by Dr. S.

*Liver.*—Enlarged in three cases, and in one of them to a great degree, in the others it was of natural or moderate size. The consistence of the organ appears to have been generally diminished, being flabby, or softened, or both, in four cases, a little soft in a fifth, and moderately firm, but still readily penetrated by the finger in a sixth; in the seventh the consistence is not mentioned.

The colour was nearly the same in every case, but very different from natural. In most of the cases the liver is described as being of the colour of bronze, or a mixture of bronze and olive, in one as a dull lead colour externally, internally bronzed with a reddish shade; in another as between a brown and an olive, the latter predominating; and finally, as a pale slightly greenish lead colour, with a tinge of brown, in one instance. Few things are more difficult than a description of colour. The most correct idea of that before us would perhaps be conveyed by stating its predominant character, the same in every case, to be a mixture of gray and olive, the natural reddish brown being entirely extinct, or only faintly to be traced.—This alteration existed uniformly or nearly so throughout the whole extent of the organ, except in a single instance, where a part of the left lobe was of the natural reddish brown hue. As the alteration of colour pervaded both substances, the two were frequently blended together, and the aspect of the cut surface remarkably uniform. In one case, however, there was a marked distinction of colour, the olive being predominant in the parenchyma, the brown in the acini. Of the four cases in which these characters are mentioned, the cut surface is described as smooth in three, of a shagreened appearance, and rough in the left lobe, in the fourth. This last character was evidently dependent upon hypertrophy of the lighter coloured substance, which existed also in another instance, both cases, however, being examples of a very protracted form of the disease.

The nature of the lesion of the liver above described, characterized essentially by a peculiar alteration of colour, is not easily determined. That it is the result of inflammation will hardly be contended, and even if attended with congestion, (which I think very doubtful,) this could not account for it, as congestion is frequently present in other diseases where no such alteration of colour is observable, and where, on the contrary, its effect is to produce a deeper red. Some, perhaps, will look upon it as dependent upon the infiltration of bile into the tissue of the organ, but still it will at once be perceived that this presupposes a peculiar alteration of the bile and liver, inasmuch as the appearance presented is not found in other diseases, at least so far as I am aware. In saying that this lesion is found in no other disease, I wish to be understood as excepting those cases of pernicious and other intermittents, which prove fatal in the early stage, or before giving rise to well developed cirrhosis, abdominal effusion, &c. Indeed I think it highly probable that the same alteration of the liver will be found to exist in intermittents which thus prove fatal, an opinion confirmed by the case last detailed. (Case III.) In speaking, therefore, of this alteration being peculiar to remittent fever, I wish to be distinctly understood as not excluding intermittent fever, which in my opinion is essentially the same disease.

The lesion in question, then, being peculiar to the disease before us, and the only one which is so, (all the other lesions being common to it and other diseases,) and at the same time being found, as already observed, in every case, we are obliged to admit that it constitutes its essential anatomical characteristic, or at least that such is the conclusion to be derived from the cases before us. Their number, I am aware, is insufficient to establish such a point conclusively, and it therefore remains for future observers to determine whether or no the lesion we have described belongs to the disease under all circumstances. That such will be found to be the case, I confess, seems to me very probable, when I recollect that the cases we have been examining were distributed over three successive seasons, and originated, not in a single locality, but in different and widely separated places, and also that by a reference to the description of authors, it is apparent that a similar condition of the liver has been frequently observed by them, without, however, attracting that attention which it seems to me it demands. Thus we find it stated, in a dissertation on remittent fever, recently published by Dr. Shapter,* and composed no doubt after a comparison of the description of various authors, that "the liver is found enlarged, injected, and softened in structure, and is generally of a *dark*, sometimes of a *gray* colour."

It has been already mentioned that the characteristic alteration of the liver was, in two protracted cases, combined with a general development of the acini or lighter coloured substance.

The terminating remarks, with the extracts we have already given, include that portion of the article which will be interesting to our readers. The remarks of the author that the alteration of the liver is not the cause of most of the symptoms, receive our unqualified assent, and place the lesion in its true point of view.

Let us now observe, in conclusion, that, of the various lesions described in the foregoing pages, two only were constant or observed in every case, viz. that of the spleen and the peculiar alteration of the liver. We might perhaps include amongst the constant lesions, the development of the glands of Brunner in the duodenum, but it may admit of doubt whether these glands were really morbidly enlarged in every case, although, at the same time, their frequent enlargement and uniform distinctness constitute a striking peculiarity of the disease. Of those which were not constant, the inflammation of the stomach is unquestionably most worthy of attention, both on account of its great frequency and the importance of the organ itself. The lesion of the spleen, though constant, was similar in appearance to that found in other diseases. It was remarkable, however, for the uniformly great degree to which it was carried, and for being frequently accompanied during life with pain on pressure in the left hypochondrium, a circumstance not usually accompanying enlargement and softening of the organ in other acute affections. The liver, on the other hand, was the seat of a morbid change not only present in every case, but of a character not met with in other diseases, and as it was

* See Tweedie's Library of Practical Medicine, vol. I.

the only lesion which offered these two conditions, the conclusion is obvious that it constituted the essential anatomical characteristic of the disease, as it presented itself to our observation. As the number of cases were small, such a conclusion when viewed as a general proposition, applicable to remittent fever universally, can only be regarded as the indication of a probable truth, the full confirmation of which requires the analysis of a more extended series of observations, conducted after the same manner. I have already given my reasons for supposing that the lesion in question will probably be found to be uniformly characteristic of the disease, and also for believing that even if such should not be found to be the case, it still had strong claims upon our attention, and shall not repeat them here. It is a circumstance particularly worthy of note that the only two fevers in which yellowness of the skin is found as a principal symptom, should also be the only ones characterised by any remarkable alteration of the liver, and this consideration is certainly calculated to give to the latter additional interest and importance. I ought perhaps here to mention that I was made acquainted with the conclusions of M. Louis in reference to the anatomical characteristic of the yellow fever of Gibraltar, some years before the publication of his work on that subject, as he kindly favoured me with a perusal of the manuscript, which he placed in my hands for that purpose shortly before my leaving Paris in the year 1834. To this circumstance I am no doubt much indebted for having my attention especially directed to the condition of the liver, particularly as the very first fatal case which occurred at the Pennsylvania Hospital, after my entrance upon the duties of my station, bore a strong resemblance in many of its symptoms to yellow fever. Admitting that the lesion of the liver, which I have described, really constitutes the essential anatomical characteristic of remittent fever, are we to look to this organ as the primary seat of the disease? To this, I think, we must answer in the negative, at least so far as our information at present extends, because, in the first place, we have no evidence that the lesion is present at the very commencement of the disease, although it is highly probable that it exists at a very early stage; and, in the second, it is impossible, even if it were so, to explain by its means all the early symptoms, or, in other words, to trace them to it as to their source.—In short, it seems to me, that here, as well as in typhoid and yellow fever, the characteristic anatomical lesions are to be regarded, like the pustules of small pox, as consequences and not as causes of the affections which they severally characterize, and that we must look for the latter in some morbid condition other than what is observed in the solid organs. It is to be hoped that the investigations now going on in reference to the alterations of the blood may throw some light upon this obscure subject.

Of the causes of death but little need be said. In some cases the condition of the organs afforded a sufficient explanation of it, especially in those two, where, in addition to the lesions generally met with, there was either extensive ulceration of the large intestine, or thinning and softening of the mucous membrane of the small intestine with partial peritonitus. In others, again, the lesions of the solid viscera do not appear to be sufficient, but, as the blood was altered in every case in which its condition was noted, we may reasonably conclude that the changes in this fluid played an important part in bringing about the fatal termination.

